# Gold, Gold-Beating, Compounds and Alloys

**Published:** 1853-04

**Authors:** R. N. Wright


### ARTICLE IV.

*Gold, Gold-beating, Compounds and Alloys.* By Professor R. N. WRIGHT, M. D.

(Continued from page 248.)

*Protochloride of Gold*—(au. + cl.)—The best process for the preparation of this substance is, to expose to the action of heat, the higher chloride, viz. the perchloride, by means of a sand bath, or any ot her method of applyingheat, which can be properly regulated.

Place the substance in a dish of Berlin porcelain, and agitate frequently, continuing the process until chlorine ceases to be evolved. A saline mass will now be found having a whitish appearance, and which should be insoluble in water; if, however, any part of the perchloride should remain undecomposed, that part will be soluble in water.

The only precaution necessary for its preservation is, that it be kept dry; for, as soon as it comes into contact with atmospheric moisture, or water derived from any other source, it becomes gradually converted into perchloride, attended with the deposition of metallic gold, and the process is much accelerated, if water at 212° F. comes into contact with it.

*Perchloride of Gold.*—(Au. + cl. 3.)—Probably the best method which can be adopted for the preparation of this substance, is to expose to the action of heat, finely divided gold in contact with chlorine gas. This gives us a substance of a fine yellow color. We may dissolve gold in "aqua regia," (nitro-hydrochloric acid,) and obtain the chloride by subsequent evaporation—the result, being a quantity of orange colored, deliquescent prismatic crystals, which are readily decomposed by heat into protochloride, and (if the heat be continued sufficiently) pure gold. Sulphuric acid, of standard strength, added to a saturated solution of this compound, has the effect of producing a compound which some chemists have called "auhydrous chloride of gold." By any process which may be used for its preparation, we have a substance which has a peculiar taste somewhat analogous to that of certain unripe fruits, and passing through several shades of color, from red to yellow, when dissolved in water; a saturated neutral solution, being red, while on the other hand, if not neutral or dilute, inclining to yellow, (especially if acid be present.)

*Purple of Cassius.*—For the following able process for preparation of the "purple of cassius," now so extensively used in dentistry, we are indebted to the able work of Prof. Graham: "When protochloride of tin is added to a dilute solution of gold,a purple-colored powder falls,which has received that name. It is obtained of a finer color when protochloride of tin is added to a solution of perchloride of iron, until the color of the liquid has a shade of green, and adding this liquid drop by drop, to a solution of perchloride of gold, which is free from nitric acid, and very dilute. After twenty-four hours, a brown powder is deposited, which is, in a small degree, transparent and purple red, by transmitted light. When dried and rubbed to powder, it is of a dull blue color. Heated to redness, it loses a little water, but no oxygen, and retains its former appearance. If washed with ammonia, on the filter, while still humid, it is dissolved, and a purple liquid passes through, which rivals the hyper-manganate of potash in beauty. From this liquid, the coloring matter very gradually separates, weeks elapsing before the upper strata of the liquid become colorless, but it is precipitated more rapidly when heated in a close vessel between 140° and 180°. The powder of cassius is insoluble in solutions of potash and soda. It may also be formed by fusing together 2 parts of gold, 3½ parts of tin, and 15 parts of silver, under borax, (to prevent the oxidation of the tin,) and treating the alloy with nitric acid to dissolve out the silver; a purple residue is left containing the tin and gold, that were employed."

The latter process will under certain circumstances be found the most practicable, requiring less accuracy in chemical manipulation than the other. Prof. Graham goes on to say, "the powder of cassius is certainly, after ignition, a mixture of peroxide of tin and metallic gold, from which the last can be dissolved out by 'aqua regia,' while the peroxide of tin is left; and the last mode of preparing it, favors the idea that its constitution is the same before ignition. But its property to dissolve in ammonia, and the fact that mercury does not dissolve out gold from the powder when properly prepared, appear to me, to be conclusive against that opinion. The proportions of its constituents vary so much, that there must be more than one compound, or more likely the coloring compound combines with more than one proportion of the peroxide of tin. Berzelius proposes the theory, that the powder of cassius, may contain the true protoxide of gold, combined with the deutoxide of tin, ($Au\ 0+Sn_2\ 0_3$)—a kind of combination containing an association of three atoms of metal, which is exemplified in black oxide of iron, spinell, gahnite, franklinite and other minerals, and which we have repeatedly observed, to be usually attended, with great stability. A glance at its formula, shows how readily the powder of cassius, as thus represented, may pass into gold and peroxide of tin; ($Au\ 0+Sn_2 0_3$)=($Au+2\ Sn\ 0_2$.) The existence of a purple oxide of gold ($Au+0$,) is not established; but, it is probably the substance formed, when a solution of gold, is applied to the skin or nails, and which dies them purple. Paper, colored purple by a solution of gold, becomes gilt, when placed humid in phosphuretted hydrogen gas, which reduces the gold to the metallic state."

*Fulminating Gold*, or aurate of ammonia is formed whenever to a tolerably concentrated solution of chloride of gold, which has been diluted with water to the extent of 2½ or 3 parts, aqua ammonia is added. When precipitation has ceased, we have in the glass, a mass bearing a yellow color inclining to brown, which is best secured by throwing the contents of the glass on a filter, washing it carefully with water, and drying by means of a gentle heat; the heat used in the process not being allowed to exceed 212°, a temperature under 300° being sufficient to produce explosion, which is attended sometimes with very unpleasant results to the operator. This substance will also explode by friction with resisting bodies, a blow with a hammer or by the electric spark. A process differing from the above and which is said to produce a substance which is more powerfully fulminating, is the following: digest peroxide of gold with aqua ammonia until a dark olive colored mass is produced, and dry it carefully, observing the same precautions as in the other instance.

*Alloys.*—For the following elaborate essay upon the subject of alloys with gold, we are indebted to Brande, and it is so complete in all its parts, and so nearly perfect, that we have determined to insert it without alteration. He says—"The alloys of gold with *potassium* and *sodium*, have not been examined. With *manganese*, it forms a gray brittle alloy, hard, somewhat ductile and of great lustre. Mr. Bingley found the proportion of manganese, to form from one-eighth to one-ninth of the compound: it was obtained by heating gold with oxide of manganese which had been repeatedly mixed and burned with olive oil. Cupellation with lead, separated the manganese. With *iron* the alloy is malleable and ductile and harder than gold; its color dull white, and its specific gravity, when containing 11 gold + 1 of iron, 16.885. The metals expand by union; so that, supposing their bulk before combination to have been 1000, after combination, it is 1014.7. With *zinc*, the compound, is brittle and brass colored, specific gravity, when containing 11 gold + 1 zinc, 16.937. The metals contract a little in uniting, the original bulk being 1000, that of the alloy is 997. The brittleness continued, when the zinc was reduced to the one-sixtieth of the alloy. The fumes of zinc in a furnace containing fused gold, make it brittle; and according to Hellot, when an alloy of 1 of gold and 7 of zinc is ignited in a crucible, the whole of the gold sublimes with the oxide of zinc. *Tin* formed a whitish alloy, brittle when thick, but flexible in thin pieces; specific gravity, when constituted of gold 11 + tin 1, 17.307; bulk before fusion 1000, after fusion 981; so that there is considerable contraction. The old chemists, called tin "diabolus metallorum," from its property of rendering gold brittle; but Mr. Bingley's experiments, quoted by Mr. Hatchett, show that one-sixtieth of tin does not render gold brittle; indeed, Mr. Alchorne's experiments (Phil. Trans. 1784,) showed that gold might be rolled and coined, when containing only one thirty-seventh of tin. At a cherry red heat, Mr. Bingley found that this alloy became dark colored, and disintegrated from the loss of the tin. Mercadien obtained the "purple of cassius," by digesting the alloy, rich in tin, in nitric acid: hydrochloric acid dissolves the tin and leaves finely divided gold. The alloy of gold with *cadmium*, has not been examined. Mr. Hatchett, obtained a dull-yellow alloy with 11 gold + 1 *cobalt*, the density of which was 17.112. The bulk of the metals before fusion being 1000, became 1001 after fusion. When the cobalt was below $\frac{1}{6}$th of the mass, the alloy was somewhat ductile. With gold and *nickel*, he obtained a brittle brass colored alloy, when in the proportions of 11 to 1. The density of the gold being 19.172, and of nickel 7-8, that of the alloy was 17.068; and the bulk of the metals before fusion being 1000, was increased after fusion to 1007. With *copper*, gold forms a ductile alloy of a deeper color, harder, and more fusible than pure gold: this alloy, in the proportion of 11 gold to 1 copper, constitutes standard gold; its density is 17.157, being a little below the mean, so that the metals slightly expand on combining. One troy pound of this alloy is coined into $46\frac{29}{40}$ sovereigns, or 20 troy pounds, into 934 sovereigns and a half. The pound was formerly coined into 44 guineas and a half. The standard gold of France consists of 9 parts of gold and 1 of copper. The alloy of gold with *lead* is very brittle when that metal only constitutes one-1920th of the alloy: even the fumes of lead destroy the ductility of gold; the specific gravity of an alloy of 11 gold + 1 lead is 18.080; and 1000 parts become 1005. A remarkable fact in respect to this alloy is, that its specific gravity diminishes to a certain extent, as the proportion of lead diminishes, and is at its maximum when the lead amounts only to one-96th part, the quantity of gold remaining the same, and the deficiency being made up with copper; the following table exhibits this:—

| Metals. | Grains. | Specific gravity of alloy. | Bulk before union. | Bulk after union. | Expansion. |
|---|---|---|---|---|---|
| Gold,<br>Lead, | 442<br>38 | 18.080. | 1000 | 1005 | 5 |
| Gold,<br>Copper,<br>Lead, | 442<br>19<br>19 | 17.765. | 1000 | 1006 | 6 |
| Gold,<br>Copper,<br>Lead, | 442<br>30<br>8 | 17.312. | 1000 | 1022 | 22 |
| Gold,<br>Copper,<br>Lead, | 442<br>34<br>4 | 17.032. | 1000 | 1035 | 35 |
| Gold,<br>Copper,<br>Lead, | 442.<br>37.5<br>0.5 | 16.627. | 1000 | 1057 | 57 |
| Gold,<br>Copper,<br>Lead, | 442.<br>37.75<br>0.75 | 17.039. | 1000 | 1031 | 31 |

Gold and *antimony* form a brittle yellow compound: Mr. Hatchett found gold made standard by antimony to be of a pale dull color, very brittle and fine grained, and its density 16.929, the bulk of the metals being reduced by combination from 1000 to 987. When the antimony only formed one-1920th of the mass, it was still brittle, and the fumes of antimony in the neighborhood of fused gold destroy its ductility. A standard alloy of gold with *bismuth* was brittle, fine grained, and of a greenish-yellow color: its density 18.038; the bulk of the metals being reduced from 1000 to 988. The color of the alloy became nearly that of gold when the bismuth formed one-60th, but it remained brittle with only one-1920th; the fumes of bismuth also destroy the ductility of gold which has been fused in the same furnace.

*Uranium*, *titanium* and *cerium* have not been examined in their relations to gold. The alloy of gold and *tellurium* exists native. *Arsenic* and gold are not easily combined, in consequence of the volatility of the former metal. Mr. Hatchett added 453 grains of arsenic to 5,307 of fused gold, and having stirred the mixture poured it into an iron mould; only 6 grains were retained, so that the alloy contained one-885th of arsenic; it had the color of gold, and was brittle; by suspending gold in the fumes of arsenic, he obtained a brittle fusible alloy of a gray color. *Molybdenum* and gold form a black, brittle compound. *(Hielm Crell's Annals, iii.)* The action of gold and *chromium*, has not been examined. Little is known of the compound of gold with *tungsten*. "When 100 parts of gold, 50 of tungstic acid, and a quantity of charcoal-powder, were strongly heated in a covered crucible, complete fusion did not take place; the button weighed 139 grains; by cupellation with lead the gold was reduced to its original purity." *Mercury* and gold combine readily at common temperatures, and more rapidly when heated, the mercury taking up a considerable proportion of gold without loss of fluidity; when rich in gold, the amalgam is of a buttery consistence, and may be separated from the more liquid compound by pressure through leather; it then consists of 2 parts of gold and 1 of mercury; the amalgam used for gilding bronze, contains about one-eighth of gold. *Silver* and gold appear to combine readily in all proportions, when the fused metals are stirred together; but, that there is a tendency to definite combination, appears from Homberg's experiments, who kept equal parts of gold and silver in fusion for a quarter of an hour, and found on breaking the crucible that the ingot consisted of two distinct portions; the uppermost being pure silver, and the lowermost gold, combined with one-sixth of silver; the density of this alloy is a little below the mean, and the color of gold is rendered paler by a twentieth part of silver. All the gold at present coined, is alloyed with copper only; previous to the year 1826, the alloy consisted in part of silver, hence the paler color of the sovereigns and half sovereigns of former coinages; an alloy composed of equal parts of silver and copper furnishes the best addition to gold for the purpose of coinage, and it is to be regretted that this requisite portion of silver is not made part of the *value* of the coin, by which the system of melting down our gold coin, for the purpose of extracting its silver would be prevented, and the perfection and facility of coinage ensured. To separate the silver from the gold, the alloy is melted with great excess of silver, granulated, and boiled in sulphuric acid, (in vessels of platinum,) by which the silver is oxidized and converted into sulphate of silver, and the metallic gold remains in the form of an insoluble black powder, which is afterwards collected, washed, and fused into a button or ingot. In the same way, the small quantity of gold contained in silver coin, which used to pass unheeded, is extracted by sulphuric acid; the recently coined silver, will accordingly be found destitute of those traces of gold which are contained in our coin of a date anterior to 1826, and in Spanish dollars, and other foreign silver. When gold and silver are parted by the action of nitric acid, it is necessary, as in the case of sulphuric acid, that the silver should be in great excess; it is otherwise partially protected from the solvent powers of the acid. Mr. Johnson, (the eminent refiner,) tells me that not only are small portions of silver volatilized, or in some way carried off by the nitrous vapors, but that gold may also be detected in the condensed fumes. In his parting furnaces, the nitrous fumes are carried downwards so as to pass through the fire, and traces of the precious metals, are found in the cinders and ashes."

The above extract is taken from Brande, page 978, and headed "Alloys of Gold." The following table of "Weights, value, &c., of British gold coin," is from the same author.

### *Table of the Weights, Value, &c., of British Gold Coin.*

| Number of pieces in the pound weight. | | Standard weight of each piece. oz. dwt. grs. | Fine gold in each piece. oz. dwt. grs. | Current weight of each piece not to pass below. oz. dwt. grs. | Allowance for current wear. grs. | Remedy by indenture in the lb. Weight. gr. | Remedy by indenture in the lb. Fineness. gr. | Working remedy for Monayer's by mint regulations. On each piece, above or below standard. gr. | Working remedy: Heaviest piece. oz. dwt. grs. | Working remedy: Lightest piece. oz. dwt. grs. | By mint regulations the bank must receive if not heavier than. dwt. grs. | Legal tender. | Standard weight in troy grains. gr. |
|---|---|---|---|---|---|---|---|---|---|---|---|---|---|
| 178 | ¼ guineas. | 0 1 8.35 | 0 1 5.66 | 0 1 8 | .35 | 40 | 40 | .22 | 0 1 8.584 | 0 1 8.134 | | To any amount. | 32.359 |
| 133½ | ⅓ ditto | 0 1 19.14 | 0 1 15.55 | 0 1 18 | 1.14 | " | " | .29 | 0 1 19.445 | 0 1 18.846 | | | 43.146 |
| 89 | ½ ditto | 0 2 16.71 | 0 2 11.32 | 0 2 16 | 71 | " | " | .44 | 0 2 17.168 | 0 2 16.269 | | | 64.719 |
| 44½ | Guineas. | 0 5 9.43 | 0 4 22.65 | 0 5 8 | 1.43 | " | " | .89 | 0 5 10.337 | 0 5 8.539 | | | 129.438 |
| 22¼ | 2 ditto | 0 10 18.87 | 0 9 21.30 | 0 10 16 | 2.87 | " | " | 1.79 | 0 10 20.674 | 0 10 17.078 | | | 258.876 |
| 9 wanting ½ guinea. | 5 ditto | 1 6 23.19 | 1 4 17.25 | 1 6 16 | 7.19 | " | " | 4.49 | 1 7 13.685 | 1 6 18.697 | | | 647.191 |
| 93 9/20 or 1869 in 20 lbs. | ½ sovereigns. | 0 2 13.63 | 0 2 8.500 | 0 2 13.125 | ½=.512 | 12 | 15 | .128 | 0 2 13.765 | 0 2 13.508 | 2 13 { 803=1/6 gr. ab. stan. | | 61.163 |
| 46 29/40 or 934 & ½ in 20 lbs. | Sovereigns. | 0 5 3.27 | 0 4 17.001 | 0 5 2.5 | ¾= .774 | " | " | .256 | 0 5 3.531 | 0 5 3.017 | 5 3 { 607=⅓ gr. ab. stan. | | 123.274 |
| 23¼ & 9/80 | £2 pieces. | 0 10 6.54 | 0 9 10.003 | 0 10 5 | 1.548 | " | " | .513 | 0 10 7.062 | 0 10 6.035 | | | 246.548 |
| 9 & 96/200 | £5 pieces. | 1 5 16.37 | 1 3 13.008 | " | " | " | " | 1.284 | 1 5 17.656 | 1 5 15.088 | | | 616.372 |

The assays of gold are reported in carats and grains ; the lowest is ⅛ ct. gr.=7½ troy grains.
Deliveries of coin are made in journey-weights, each journey=15 lbs. troy, in value, £700 17*s.* 6*d.*

There remain now to be described some of the compounds formed by combinations of gold and non-metallic elements, and that class of compounds described by Bonsdorff and called "auro-perchlorides."

*Phosphuret of Gold.*—This substance may be procured by igniting phosphorus in contact with gold in a state of comminution; the process to be conducted in an apparatus of narrow aperture, deprived of air and so constructed that it cannot gain access to it. A tube answers the purpose perfectly well.

Another method of producing phosphuret of gold, is to drop the solution of gold into excess of phosphuretted hydrogen, metallic gold being the more common precipitate when the phosphuretted hydrogen is not in excess.

We have produced by either process, a substance decomposed by action of heat in contact with atmospheric air—which has a grayish color, considerable metallic lustre and which consists of one atom of each of its constituents, (gold 1, phosphorus 1.)

*Iodide of Gold* is most easily obtained by putting together chloride of gold and iodide of potassium, washing subsequently to get rid of any excess of iodine. The result of the process is a yellow mass, which is easily decomposed by heat, which will not dissolve in water below 60° F. and which consists of (gold 1 part, iodine 1 part.)

*Bromide of Gold.*—The union of bromine and gold, forming the bromide of gold, produces a substance which is crytallizable, of a most intense brown color, and which consists of (bromine 1 part, gold 1 part.)

*Percyanuret of Gold.*—When cyanuret of potassium is added to cloride of gold—the result is a precipitate having a yellow color. It is necessary, however, not to let the cyanuret be in excess, for the precipitate is soluble in the precipitant, and an acid will have to be added in such cases, in order to re-obtain it.

*Sulphocyanuret of Gold*, is obtained by a process similar to the above, using only sulphocyanuret of potassium with the chloride of gold, instead of the cyanuret; and the result is a pink precipitate.

*Sulphuret of Gold.*—In order to prepare this substance, it is only necessary to pass a current of sulphuretted hydrogen gas through perchloride of gold dissolved in water. The result is a black precipitate, and, as is the case with many sulphurets so formed, heat will decompose it and the result of decomposition will be sulphur and gold.

By varying the above process, we may obtain a soluble compound which has been described as the sulphuret of gold and potassium. In order to prepare it, we must melt together potassa, gold and sulphur.

*Auro-Perchlorides.*—Brande says of these compounds, "under this term are comprehended the compounds described by Bonsdorff (Aun. de Ch. et Ph., xliv,) and others, in which the chloride of gold is combined with certain electro-positive chlorides, such as those of the alkaline bases: these consist of one atom of terchloride of gold, and one atom of the other chloride, and may be formed of their respective chlorides, in such proportions; some of them have been long known; they mostly form prismatic crystals, and include water of crystallization. It is in consequence of the formation of these soluble double salts, that the solution of chloride of gold in hydrochloric acid yields no precipitates with the alkalies even when added in excess. Different auro-chlorides, obtained by adding salts of potassa, soda, ammonia and other bases, to the chloride, are employed in gilding copper trinkets, buttons and other articles, under Mr. Elkinton's patent."

The author of the above goes on to describe several auro-perchlorides, as follows:

"Auro-perchloride of ammonia is formed by dissolving the component salts in atomic equivalents, and evaporating; it crystallizes in efflorescent acicular prisms, soluble in water and alcohol, and containing:

| | | | |
|---|---|---|---|
| Hydro chlorate of ammonia, . . | 1 | 54 | 13.6 |
| Perchloride of gold, . . . . . | 1 | 308 | 77.4 |
| Water, . . . . . . . . | 4 | 36 | 9.0 |
| | — | —— | —— |
| Auro-perchloride of gold, (crystallized,) | 1 | 398 | 100.0" |

"*Auro-Perchloride of Potassium.*—This salt may be obtained by evaporating mixed solutions of chloride of potassium and perchloride of gold in yellow four-sided prisms, slightly efflorescent, and which, dried at 212°, lose their water of crystallization; at a higher temperature chlorine is evolved, and the salt fuses into a dark-brown liquid, which probably contains protochloride of gold, but which water and dilute hydrochloric acid, resolve into the original salt, chloride of potassium and metallic gold being separated. The components of the crystallized double chloride are, according to Bondsdorff,

| | | | |
|---|---|---|---|
| Chloride of potassium, . . . | 1 | 76 | 17.8 |
| Perchloride of gold, . . . . | 1 | 308 | 71.7 |
| Water, . . . . . . | 5 | 45 | 10.5 |
| Crystallized auro-perchloride of potassium, | 1 | 429 | 100.0 |

"Auro-perchloride of sodium, forms four-sided prisms, permanent in the air, and easily fusible in their water of crystallization; they contain,

| | | | |
|---|---|---|---|
| Chloride of sodium, . . . . | 1 | 60 | 14.5 |
| Perchloride of gold, . . . . | 1 | 308 | 74.4 |
| Water, . . . . . . | 5 | 45 | 11.1 |
| Crystallized auro-chloride of sodium, | 1 | 413 | 100.0 |

"Auro-perchlorides of lithium, calcium, barium, strontium, magnesium, manganese, zinc, cadmium, cobalt and nickel, have also been examined by Bonsdorff: they form hydrated prismatic salts, generally similar to the preceding."

Having given an outline of the subjects embraced under the caption of this article, and having made no stinted use of the standard authorities whose researches would in any manner assist in its completion, we propose to conclude, with a process for "electro-gilding," (as it is called,) selecting that of Mr. Smee, so justly famous in this department of science.

We subjoin the process of electro-gilding by Mr. Smee, which he considers best. After enumerating most of the other methods in use, he thus speaks of them, (Art. 195:) "All these methods appear to me very objectionable, and a far more manageable mode of gilding, is to take three, four, or more of my batteries, and charge them with very dilute sulphuric acid, At the two extremities, attach *platinum* wires, one connecting the zinc of the battery, with the metal to be gilt, the other forming the anode or electro-positive pole in the decomposition cell. The muriate of gold must be diluted and slightly acid. Into this, the platinum wire, which is connected with the silver of the batteries is to be placed; but, it must be immersed only to a very trifling depth. Having thus completed these arrangements, the object to be gilt is made to communicate with the zinc of the battery, and immediately, on its being placed in the solution, the gold will begin to be precipitated.

Every portion of the object on which we are desirous not to have the layer of gold, must be coated with tallow, wax, or any other non-conducting substance, which will prevent any deposit from taking place on those parts. In this way, an object may be coated up to any desired limit, or upon any circumscribed parts of its surface, as, for example, drawing or writing thereon. The rapidity of the process may be regulated to the greatest nicety by placing more or less of the positive wire in the solution, by which means, as in other cases, the quantity of electricity passing may be regulated with the utmost precision.

To conduct this elegant process with the greatest economy of time, the quantity of electricity should be so regulated, that the hydrogen is kept just below the point of solution from the negative plate; for we must always bear in mind, that the evolution of hydrogen, is attended with evil, as the precipitate will be in the finely divided state, or black powder.

During the process, particularly if the object have a rough surface, it is a good plan to remove it once or twice from the solution, and to rub it with a small quantity of whiting, and well wash it; by these means, any finely divided metal will be removed, and the gold will be precipitated in a very even manner. The color of the gold, if the precipitated layer be very thin, will be a greenish yellow, but, when thicker it will be the natural color of the pure metal. A little copper, added to the solution of gold, will cause the precipitated metal to be very red. The preceding observations apply equally to those cases, where we desire to gild platinuum, palladium, silver or gold itself."

With regard to gilding generally by galvanism, one caution should be especially observed, viz. that the article to be gilded, should not be introduced into the solution until all things are ready for the immediate completion of the circuit, and this caution is necessary from the fact, that some metals have a local action on the solution of gold, and if such an action commences, it will be scarcely possible to obtain a smooth deposit. It is best, therefore, always to commence the process with a dilute solution, and after a very thin layer has been obtained, its strength may be increased at the pleasure of the operator, to almost any extent.

It is intended to follow this article with others, so as to lay before the readers of the Journal as much practical information in relation to their metals especially useful in dentistry, as can be obtained from any source—and in making our selections, we will endeavor in all cases, (where it is practicable,) to let the author speak for himself.

The subject of the next article will be silver.

